# Cerebral Amyloid Angiopathy and Downstream Alzheimer Disease Plasma Biomarkers

**DOI:** 10.1001/jamanetworkopen.2025.8842

**Published:** 2025-05-09

**Authors:** Sung Hoon Kang, Eun Hye Lee, Young Ju Kim, Hyemin Jang, Daeun Shin, Henrik Zetterberg, Kaj Blennow, Fernando Gonzalez-Ortiz, Nicholas J. Ashton, Jihwan Yun, Hee Jin Kim, Duk L. Na, Jun Pyo Kim, Sang Won Seo

**Affiliations:** 1 Department of Neurology, Samsung Medical Center, Sungkyunkwan University School of Medicine, Seoul, Republic of Korea; 2 Department of Neurology, Korea University Guro Hospital, Korea University College of Medicine, Seoul, Republic of Korea; 3 Department of Radiology and Imaging Sciences, Indiana University School of Medicine, Indianapolis; 4 Indiana Alzheimer Disease Research Center, Indiana University School of Medicine, Indianapolis; 5 Alzheimer’s Disease Convergence Research Center, Samsung Medical Center, Seoul, Republic of Korea; 6 Department of Neurology, Seoul National University Hospital, Seoul National University School of Medicine, Seoul, Republic of Korea; 7 Department of Psychiatry and Neurochemistry, Institute of Neuroscience and Physiology, the Sahlgrenska Academy at the University of Gothenburg, Gothenburg, Sweden; 8 Clinical Neurochemistry Laboratory, Sahlgrenska University Hospital, Gothenburg, Sweden; 9 Department of Neurodegenerative Disease, UCL Queen Square Institute of Neurology, London, United Kingdom; 10 UK Dementia Research Institute at UCL, London, United Kingdom; 11 Hong Kong Center for Neurodegenerative Diseases, Hong Kong, People’s Republic of China; 12 Wisconsin Alzheimer’s Disease Research Center, University of Wisconsin School of Medicine and Public Health, University of Wisconsin-Madison; 13 Paris Brain Institute, Pitié-Salpêtrière Hospital, Sorbonne University, Paris, France; 14 Neurodegenerative Disorder Research Center, Division of Life Sciences and Medicine, and Department of Neurology, Institute on Aging and Brain Disorders, University of Science and Technology of China and First Affiliated Hospital of University of Science and Technology of China, Hefei, People’s Republic of China; 15 Institute of Psychiatry, Psychology and Neuroscience, Maurice Wohl Clinical Neuroscience Institute, King’s College London, London, United Kingdom; 16 National Institute of Health Research Biomedical Research Centre for Mental Health and Biomedical Research Unit for Dementia at South London and Maudsley NHS Foundation, London, United Kingdom; 17 Centre for Age-Related Medicine, Stavanger University Hospital, Stavanger, Norway; 18 Department of Neurology, Soonchunhyang University Bucheon Hospital, Gyeonggi-do, Republic of Korea; 19 Department of Digital Health, Samsung Advanced Institute of Health Sciences and Technology, Sungkyunkwan University, Seoul, Republic of Korea; 20 Department of Health Sciences and Technology, Samsung Advanced Institute of Health Sciences and Technology, Sungkyunkwan University, Seoul, Republic of Korea; 21 Department of Intelligent Precision Healthcare Convergence, Sungkyunkwan University, Suwon, Republic of Korea

## Abstract

**Importance:**

As amyloid-targeted therapies have become commercially available, the monitoring of cerebral amyloid angiopathy (CAA), which is an important risk factor for amyloid-related imaging abnormalities, has received increasing attention. However, comprehensive evidence on the association between Alzheimer disease (AD) plasma biomarkers and various CAA imaging markers is still lacking.

**Objective:**

To examine the association of CAA imaging markers with downstream AD plasma biomarkers in relation to amyloid-β (Aβ) uptake on positron emission tomography (PET) and whether their interplay is associated with cognitive changes.

**Design, Setting, and Participants:**

This registry-based cohort study in 25 hospitals across South Korea recruited participants aged 45 years or older who were registered between January 1, 2016, and December 31, 2023. Participants were categorized as having no cognitive impairment, mild cognitive impairment, or dementia of the Alzheimer type.

**Exposures:**

Cerebral amyloid angiopathy imaging markers assessed by magnetic resonance imaging, including cerebral microbleeds (CMBs), cortical superficial siderosis, white matter hyperintensities, lacunes, and enlarged perivascular spaces.

**Main Outcomes and Measures:**

Plasma phosphorylated tau-217 (p-tau217) was measured using a commercial assay. Glial fibrillary acidic protein (GFAP) and neurofilament light chain (NfL) were measured using a single-molecule assay on a single platform. All participants underwent amyloid PET imaging. Associations of CAA and vascular imaging markers with downstream AD plasma biomarkers were investigated using linear regression.

**Results:**

A total of 1708 participants were included (mean [SD] age, 71.2 [8.4] years; 1044 female [61.1%]). The mean (SD) follow-up period was 4.3 (3.1) years. Lobar CMB counts and the presence of CAA were associated with downstream AD plasma biomarkers, including p-tau217 (β = 0.12 [95% CI, 0.05-0.18] and 0.29 [95% CI, 0.12-0.47], respectively), GFAP (β = 0.07 [95% CI, 0.03-0.12] and 0.20 [95% CI, 0.09-0.31], respectively), and NfL (β = 0.07 [95% CI, 0.03-0.11] and 0.16 [95% CI, 0.06-0.25], respectively) with and without the mediation of Aβ uptake on PET (indirect effect: lobar CMBs–p-tau217, 59.8% [β = 0.07 (95% CI, 0.03-0.11)]; lobar CMBs-GFAP, 49.3% [β = 0.04 (95% CI, 0.01-0.06)]; lobar CMBs-NfL, 20.9% [β = 0.01 (95% CI, 0.01-0.03)]; CAA–p-tau217, 50.9% [β = 0.15 (95% CI, 0.06-0.24)]; CAA-GFAP, 39.2% [β = 0.08 (95% CI, 0.03-0.13)]; CAA-NfL, 19.2% [β = 0.03 (95% CI, 0.01-0.05)]). Amyloid-β uptake fully mediated the associations between cortical superficial siderosis and downstream AD plasma markers. In contrast, hypertensive arteriosclerotic vascular imaging markers, including lacunes, deep CMBs, and enlarged perivascular spaces in basal ganglia, were associated with only NfL levels (β = 0.07 [95% CI, 0.01-0.13], 0.20 [95% CI, 0.08-0.32], and 0.14 [95% CI, 0.06-0.23], respectively), regardless of Aβ uptake on PET. Finally, there were interactive associations of lobar CMBs in conjunction with p-tau217 levels (β = −0.56 [95% CI, −0.79 to −0.34]) and GFAP levels (β = −0.44 [95% CI, −0.70 to −0.17]) with annual Mini-Mental State Examination changes.

**Conclusions and Relevance:**

In this cohort study of participants with no cognitive impairment, mild cognitive impairment, or dementia of the Alzheimer type, a novel association was found among CAA imaging markers, downstream AD plasma biomarkers, and cognitive declines in relation to brain Aβ burdens. The findings emphasize the importance of understanding the clinical effects of amyloid-related imaging abnormality–like CAA imaging markers in light of upcoming amyloid-targeted therapies.

## Key Points

**Question:**

What is the association of cerebral amyloid angiopathy (CAA) imaging markers with downstream Alzheimer disease plasma biomarkers?

**Findings:**

In this cohort study of 1708 participants, CAA imaging markers were associated with increased downstream Alzheimer disease plasma biomarkers, and amyloid-β uptake on positron emission tomography mediated these associations. Additionally, lobar cerebral microbleeds, in combination with phosphorylated tau-217 or glial fibrillary acidic protein, were synergistically associated with cognitive changes.

**Meaning:**

These findings suggest that beyond the common plasma biomarkers, developing new approaches to detect CAA and monitor amyloid-related imaging abnormalities may be essential for the new era of amyloid-targeted therapies.

## Teaser

This cohort study examines the association of cerebral amyloid angiopathy markers with downstream Alzheimer disease plasma biomarkers and whether their interplay is mediated by cognitive changes.

## Introduction

Amyloid-targeted therapies, such as monoclonal antibodies targeting amyloid-β (Aβ), are emerging treatments for Alzheimer disease (AD). These therapies, however, are associated with a side effect known as amyloid-related imaging abnormalities (ARIA). Especially, ARIA-hemorrhage is characterized by cerebral microbleeds (CMBs), cerebral superficial siderosis (CSS), and macrohemorrhages.^1^ Cerebral amyloid angiopathy (CAA) refers to amyloid deposits in the walls of cerebral blood vessels, resembling ARIA-hemorrhage on magnetic resonance imaging (MRI). Considering that CAA is a major risk factor of ARIA,^2^ identifying individuals with CAA imaging markers is crucial to manage and mitigate potential adverse effects during treatment.

Both lobar CMBs and CSS are important imaging markers of CAA-related pathology and may also affect cognitive function. Moreover, it has been reported that AD and CAA often coexist in patients with a rapidly progressive form of AD.^3^ A previous study by our group showed that these CAA imaging markers had distinctive associations with cortical atrophy and cognitive functions in relation to Aβ uptake on positron emission tomography (PET).^4^ However, the association between CAA imaging markers and downstream AD processes, including tau hyperphosphorylation, neuroinflammation, and neurodegeneration other than Aβ burdens, has not been extensively studied. Understanding the association between CAA and these downstream AD processes, as well as whether their interactions are associated with cognitive changes, is crucial for monitoring the therapeutic efficacy and side effects of amyloid-targeted therapies.

Recent advancements in clinical chemistry have enabled precise quantification of AD biomarkers using plasma.^5,6,7,8,9^ The plasma Aβ_42/40_ ratio has revealed a promising discriminative performance for detecting Aβ positivity with good to excellent accuracy.^10^ Additionally, several epitopes of phosphorylated tau (p-tau),^11,12,13,14^ glial fibrillary acidic protein (GFAP),^15,16,17^ and neurofilament light chain (NfL)^18,19^ have been studied as promising plasma biomarkers for downstream AD processes, reflecting tau hyperphosphorylation, astrocytic activation, and neuronal damage, respectively. Although these downstream AD plasma biomarkers were highly correlated with Aβ uptake on PET, they also exhibited intrinsic characteristics. Specifically, p-tau217 is more correlated with tau uptake on PET even after correction for Aβ uptake, while GFAP and NfL effectively distinguished between patients with Aβ-negative non-AD dementia, who are expected to have nonspecific tissue reactions, and individuals who are Aβ negative and without cognitive impairment.^20^ Although recent studies have reported the association between CAA and these downstream AD plasma biomarkers, besides Aβ markers, these results are inconsistent.^21,22,23,24^ Furthermore, as amyloid-targeted therapies become widely available among Asian populations, and studies have shown that ARIA frequency differs between Asian and non-Hispanic White patients,^25^ it is important to identify the association among CAA imaging markers, various downstream AD plasma biomarkers, and cognitive decline in Asian patients.

In this study, we investigated whether CAA imaging markers are associated with downstream AD plasma biomarkers (p-tau217, GFAP, and NfL) based on Aβ uptake on PET. We also examined whether CAA imaging markers and downstream AD plasma biomarkers are synergistically associated with cognitive changes.

## Methods

### Study Population

This cohort study recruited participants from among those registered between January 1, 2016, and December 31, 2023, in the Korea-Registries to Overcome and Accelerate Dementia Research project^26^ in collaboration with 25 nationwide university-affiliated hospitals in South Korea (eFigure 1 in Supplement 1). The Samsung Medical Center Institutional Review Board approved this study. All participants provided written informed consent, and the data were collected according to the principles of the Declaration of Helsinki.^27^ This study followed the Strengthening the Reporting of Observational Studies in Epidemiology (STROBE) reporting guideline.

This project is a member of the worldwide Alzheimer Disease Neuroimaging Initiative. We enrolled participants aged 45 years or older who underwent Aβ PET and brain MRI and whose AD plasma biomarkers were measured. The participants were grouped as those who were cognitively unimpaired, had mild cognitive impairment, and had dementia of the Alzheimer type. The detailed criteria for these diagnoses are described in the eMethods in Supplement 1.

We excluded patients with structural lesions, including territorial infarction, brain tumors, white matter hyperintensities (WMHs) due to demyelinating disorders, vasculitis, leukodystrophy, radiation injury, and hydrocephalus on MRI. Additionally, patients with abnormal laboratory results on complete blood count, electrolyte, vitamin B12 and folate, syphilis serology, liver, kidney, and thyroid function tests were excluded.

### MRI Acquisition

All participants underwent a baseline brain MRI at the Samsung Medical Center using a 3.0T Achieva scanner (Philips Healthcare). Three-dimensional T1 turbo field echo images, fluid-attenuated inversion recovery (FLAIR), and T2-weighted gradient-echo images were acquired. Detailed protocols based on previous studies are described in the eMethods in Supplement 1.^28,29^

### Visual Assessments of CAA and Vascular Imaging Markers on MRI

The number of CMBs 10 mm or less in diameter on 20 axial slices of T2-weighted gradient-echo image MRI was evaluated.^30^ The distribution of CMBs was classified as lobar or deep regions according to the criteria proposed by Gregoire et al.^31^ The presence of CSS was defined as linear chronic blood residues in the superficial layers of the cerebral cortex.^32^ Additionally, the presence of CAA was defined as having MRI features suggestive of probable CAA based on the Boston criteria, version 2.0.^33^ White matter hyperintensity burdens in the deep subcortical and periventricular regions in FLAIR images were evaluated according to the modified Fazekas scale, as described previously.^34^ The presence of WMHs was defined as either moderate or severe depending on WMH severity. The detailed criteria for WMH severity are described in the eMethods in Supplement 1. Enlarged perivascular spaces (EPVSs) were evaluated in line with Standards for Reporting Vascular Changes on Neuroimaging criteria^35^ and rated on T2-weighted images according to a previously validated 4-point visual rating scale in the basal ganglia and centrum semiovale as follows: 0 points (no EPVSs), 1 point (≤10 EPVSs), 2 points (11-20 EPVSs), 3 points (21-40 EPVSs), and 4 points (>40 EPVSs).^36^ The presence of EPVSs was defined as EPVS points 3 and 4. The number of lacunes was evaluated according to the consensus criteria proposed by Wardlaw et al^37^ as follows: small lesions (≤15 mm and ≥3 mm in diameter) with low signal on T1-weighted images, high signal on T2-weighted images, and a perilesional halo on 80 axial slices of FLAIR images. Four experienced neurologists (E.H.L., D.S., J.Y., and 1 nonauthor) masked to participant information rated MRI findings according to these criteria.

### Aβ PET Acquisition

All participants underwent Aβ PET (^18^F-florbetaben [FBB] PET or ^18^F-flutemetamol [FMM] PET) scans using a Discovery STE PET/computed tomography scanner (GE Medical Systems). For FBB or FMM PET, a 20-minute PET scan in dynamic mode (consisting of 4 × 5-minute frames) was performed 90 minutes after an injection of a mean dose of 311.5 MBq FBB or 197.7 MBq FMM. Three-dimensional PET images were reconstructed in a 128 × 128 × 48 matrix with a 2 × 2 × 3.27–mm voxel size using the ordered subsets expectation maximization algorithm (FBB iteration of 4 and subset of 20, FMM iteration of 4 and subset of 20).

### Aβ PET Quantification Using Centiloid Values

We followed the centiloid (CL) process described by Klunk et al.^38^ There were 3 steps to obtain CL values: (1) preprocessing of PET images, (2) determination of CL global cortical target volume of interest, and (3) conversion of standardized uptake value ratio (SUVR) to CL values. To acquire CL units, we first calculated the SUVR standard using the whole cerebellum as a reference and then calculated the FBB and FMM CL standard values using the CL transformation equation derived from previous studies on FBB (FBB CL standard = 153.4 × FBB SUVR standard − 154.9)^39^ and FMM (FMM CL standard = 121.42 × FMM SUVR standard − 121.16).^40^ Detailed protocols are described in the eMethods in Supplement 1.

### Plasma Collection and Processing

We obtained 8 mL of blood from each participant, which was deposited into a 0.5 mol/L EDTA tube and mixed for 5 minutes. Plasma was extracted from the blood sample after a 10-minute centrifuge (1300*g*) and dispensed into 5 or 10 vials at a volume of 0.3 mL each. All plasma samples were kept frozen at −75 °C until analysis. The process complied with the manual for human resource collection and registration of the National Biobank of the Republic of Korea. The median interval between plasma collection and Aβ PET scans was 4 days (IQR, 0-69 days).

Frozen plasma samples were shipped at −70 °C to the Department of Psychiatry and Neurochemistry, University of Gothenburg, for analysis. These samples were thawed on wet ice and centrifuged at 500*g* for 5 minutes at 4 °C. Plasma GFAP and NfL were quantified using the Neurology 4-Plex E Kit (Quanterix Corp). Plasma p-tau217 was analyzed using the ALZpath pTau217 Assay (ALZpath, Inc).

### Longitudinal Cognitive Evaluation

A subset of participants underwent follow-up Mini-Mental State Examination (MMSE) assessments. Annual MMSE changes were calculated for each participant using linear regression on MMSE scores across all MMSE time points, with the slope of the line representing the annual MMSE change.

### Statistical Analysis

The clinical characteristics of the study participants are presented as mean (SD) for continuous variables and as frequency (%) for categorical variables. The number of lacunes, lobar CMBs, and deep CMBs were transformed to a log scale to meet the assumption of linearity. The levels of Aβ_42/40_ ratio, p-tau217, GFAP, and NfL were also transformed.

We performed linear regression analyses to investigate the association of CAA and vascular imaging markers, including natural logarithm of lobar CMBs [Ln(lobar CMBs)], the presence of CSS, the presence of CAA, the presence of WMHs, Ln(lacunes), Ln(deep CMBs), the presence of EPVSs in the basal ganglia, and the presence of EPVSs in the centrum semiovale, with AD plasma biomarkers, including p-tau217, GFAP, and NfL, after controlling for age, sex, body mass index (BMI) status, and *APOE* genotype (*APOE* ε4 carrier status). We included BMI status and *APOE* genotype as confounders because these variables may influence both CAA imaging markers and plasma biomarkers.^41^ In this study, BMI (calculated as weight in kilograms divided by height in meters squared) status was stratified as follows^42,43^: underweight (<18.5), normal weight (18.5-24.9), and obese (>25 kg/m^2^). *APOE* ε4 carriers were defined as individuals with the ε2/ε4, ε3/ε4, or ε4/ε4 genotype. *APOE* ε2 carriers were defined as individuals with the ε2/ε2 or ε2/ε3 genotype. We conducted a mediation analysis to assess whether Aβ uptake on PET mediated the association between selected CAA and vascular imaging markers and plasma biomarkers in linear regression models (*P* < .01), after adjusting for age, sex, BMI status, and *APOE* genotype. Bootstrapping was used to verify the significance of indirect effects.

To explore the combined effect of CAA and vascular imaging markers and downstream AD plasma biomarkers on longitudinal cognitive changes, we performed linear regression analyses. Each selected CAA and vascular imaging marker was included in the linear regression models to evaluate whether its interaction with p-tau217 or GFAP was associated with annual MMSE changes. Specifically, p-tau217 by CAA and vascular imaging markers or GFAP by CAA and vascular markers were used as 2-way interaction terms. We adjusted for age, sex, BMI status, *APOE* genotype, and years of education.

Significance was set at a 2-sided *P* < .05 after false discovery rate correction within the analyses per each biomarker. Missing data in each analysis were excluded from the analysis. All analyses were performed using R, version 4.3.2 (R Foundation).

## Results

### Participant Characteristics

The study included 1708 participants (mean [SD] age, 71.2 [8.4] years; 1044 female [61.1%] and 664 male [38.9%]) (Table 1). The frequencies of CAA and vascular imaging markers among all participants were as follows: 25 (1.5%) with CSS (24 [96.0%] of which were amyloid Klunk CL of ≥20), 166 (9.7%) with CAA, 391 (22.9%) with WMHs, 165 (9.7%) with EPVSs in the basal ganglia, and 444 (26.0%) with EPVSs in the centrum semiovale. The mean (SD) number of lobar CMBs was 1.7 (15.4); of lacunes, 0.3 (1.0); and of deep CMBs, 0.1 (1.2). The characteristics of patients excluded from the subsequent mediation analysis and the longitudinal cognitive change analysis are presented in eTable 1 in Supplement 1.

**Table 1.** Baseline Demographic and Clinical Characteristics of Study Population

Characteristic	Participants, No. (%)				*P* value
	Total (N = 1708)	No cognitive impairment (n = 438)	Mild cognitive impairment (n = 762)	Dementia of the Alzheimer type (n = 508)	
Age, mean (SD), y	71.2 (8.4)	71.3 (7.3)	71.8 (8.0)	70.3 (9.6)	.009
Sex					
Female	1044 (61.1)	263 (60.0)	451 (59.2)	330 (65.0)	.10
Male	664 (38.9)	175 (40.0)	311 (40.8)	178 (35.0)	
Education, mean (SD), y	11.4 (4.7)	11.9 (4.7)	11.6 (4.7)	10.7 (4.8)	<.001
*APOE* genotype					
ε3/ε3	849 (49.8)	268 (61.2)	371 (48.8)	210 (41.3)	<.001
ε4 carriers	739 (43.3)	43 (9.8)	48 (6.3)	27 (5.3)	
ε2 carriers	118 (6.9)	127 (29.0)	341 (44.9)	271 (53.3)	
BMI status					
Normal weight (18.5-24.9)	1149 (67.3)	284 (64.8)	515 (67.6)	350 (68.9)	.003
Obese (>25.0)	497 (29.1)	148 (33.8)	218 (28.6)	131 (25.8)	
Underweight (<18.5)	62 (3.6)	6 (1.4)	29 (3.8)	27 (5.3)	
Vascular risk factors					
Hypertension	800 (46.8)	212 (48.4)	376 (49.3)	212 (41.7)	.02
Diabetes mellitus	370 (21.7)	94 (21.5)	175 (23.0)	101 (19.9)	.42
CAA and vascular imaging markers					
No. of lobar CMBs, mean (SD)	1.7 (15.4)	1.4 (15.1)	1.0 (5.9)	2.9 (23.4)	.08
Presence of CSS	25 (1.5)	4 (0.9)	14 (1.8)	7 (1.4)	.43
Presence of CAA^a^	166 (9.7)	28 (6.4)	83 (10.9)	55 (10.9)	.02
Presence of WMHs	391 (22.9)	80 (18.3)	164 (21.5)	147 (28.9)	<.001
No. of lacunes, mean (SD)	0.3 (1.0)	0.3 (0.9)	0.3 (1.1)	0.4 (1.1)	.41
No. of deep CMBs, mean (SD)	0.1 (1.2)	0.1 (1.8)	0.1 (0.7)	0.1 (0.9)	.92
Presence of EPVSs in basal ganglia	165 (9.7)	38 (8.7)	84 (11.0)	43 (8.5)	.23
Presence of EPVSs in centrum semiovale	444 (26.0)	113 (25.8)	197 (25.9)	134 (26.4)	.97

Abbreviations: BMI, body mass index (calculated as weight in kilograms divided by height in meters squared); CAA, cerebral amyloid angiopathy; CMB, cerebral microbleed; CSS, cortical superficial siderosis; EPVS, enlarged perivascular space; WMH, white matter hyperintensity.

^a^ The presence of CAA was defined as having magnetic resonance imaging features suggestive of probable CAA based on the Boston criteria, version 2.0.

### Association of CAA and Vascular Imaging Makers With Downstream AD Plasma Biomarkers in Relation to Aβ Uptake

**Figure 1.**
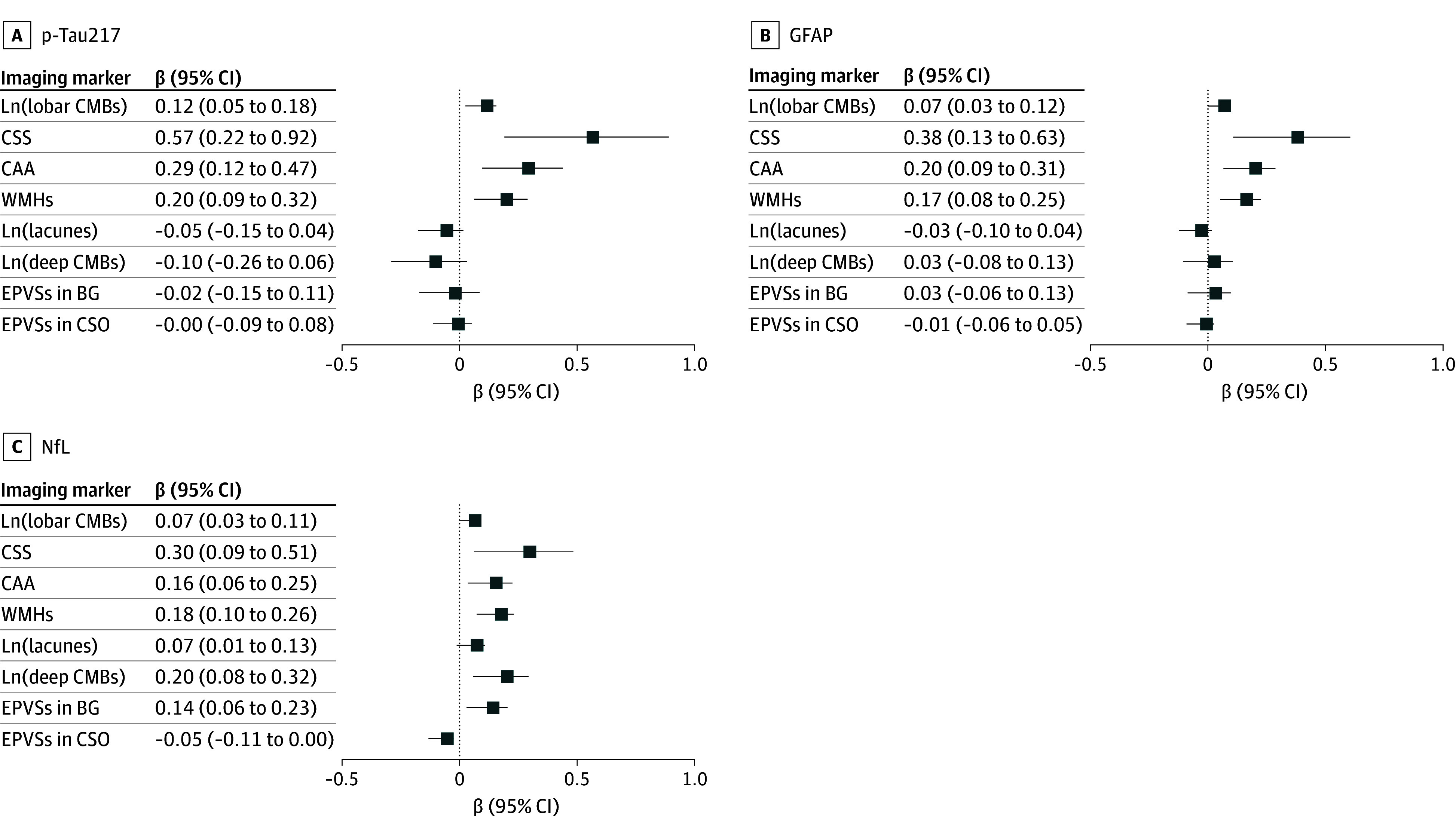
Associations of Cerebral Amyloid Angiopathy (CAA) and Vascular Imaging Markers With Alzheimer Disease Plasma Biomarkers Linear regression analyses were controlled for age, sex, body mass index status, and *APOE* genotype. The presence of CAA was defined as having magnetic resonance imaging features suggestive of probable CAA based on the Boston criteria, version 2.0. Aβ indicates amyloid-β; BG, basal ganglia; CMB, cerebral microbleed; CSO, centrum semiovale; CSS, cortical superficial siderosis; EPVS, enlarged perivascular space; Ln, natural logarithm; NfL, neurofilament light chain; p-tau217; phosphorylated tau-217; WMH, white matter hyperintensity.

The number of lobar CMBs was associated with increased p-tau217 levels (β = 0.12 [95% CI, 0.05-0.18]), GFAP levels (β = 0.07 [95% CI, 0.03-0.12]), and NfL levels (β = 0.07 [95% CI, 0.03-0.11]) (Table 2; Figure 1). The presence of CSS, CAA, and WMH were associated with increased p-tau217 levels (β = 0.57 [95% CI, 0.22-0.92], 0.29 [95% CI, 0.12-0.47], and 0.20 [95% CI, 0.09-0.32], respectively), GFAP levels (β = 0.38 [95% CI, 0.14-0.63], 0.20 [95% CI, 0.09-0.31], and 0.17 [95% CI, 0.08-0.25], respectively), and NfL levels (β = 0.30 [95% CI, 0.09-0.51], 0.16 [95% CI, 0.06-0.25], and 0.18 [95% CI, 0.10-0.26], respectively). The numbers of lacunes, deep CMBs, and EPVSs in the basal ganglia were associated only with NfL levels (β = 0.07 [95% CI, 0.02-0.13], 0.20 [95% CI, 0.08-0.32], and 0.14 [95% CI, 0.06-0.23], respectively).

**Table 2.** Associations of CAA and Vascular Imaging Markers With Alzheimer Disease Plasma Biomarkers

Imaging marker	p-Tau217		GFAP		NfL	
	β (95% CI)^a^	*P* value	β (95% CI)^a^	*P* value	β (95% CI)^a^	*P* value
Ln(lobar CMBs)	0.12 (0.05 to 0.18)	<.001	0.07 (0.03 to 0.12)	<.001	0.07 (0.03 to 0.11)	<.001
CSS	0.57 (0.22 to 0.92)	<.001	0.38 (0.13 to 0.63)	.001	0.30 (0.09 to 0.51)	.004
CAA^b^	0.29 (0.12 to 0.47)	<.001	0.20 (0.09 to 0.31)	<.001	0.16 (0.06 to 0.25)	<.001
WMHs	0.20 (0.09 to 0.32)	<.001	0.17 (0.08 to 0.25)	<.001	0.18 (0.10 to 0.26)	<.001
Ln(lacunes)	−0.05 (−0.15 to 0.04)	.32	−0.03 (−0.10 to 0.04)	.61	0.07 (0.01 to 0.13)	.01
Ln(deep CMBs)	−0.10 (−0.26 to 0.06)	.27	0.03 (−0.08 to 0.13)	.69	0.20 (0.08 to 0.32)	<.001
EPVSs in basal ganglia	−0.02 (−0.15 to 0.11)	.91	0.03 (−0.06 to 0.13)	.61	0.14 (0.06 to 0.23)	<.001
EPVSs in centrum semiovale	0.00 (−0.09 to 0.08)	.92	−0.01 (−0.06 to 0.05)	.84	−0.05 (−0.11 to 0.00)	.05

Abbreviations: CAA, cerebral amyloid angiopathy; CMB, cerebral microbleed; CSS, cortical superficial siderosis; EPVS, enlarged perivascular space; GFAP, glial fibrillary acidic protein; Ln, natural logarithm; NfL, neurofilament light chain; p-tau217, phosphorylated tau-217; WMH, white matter hyperintensity.

^a^ The β-value was obtained by linear regression analyses of the association of each vascular marker, including WMHs, Ln(lacunes), Ln(deep CMBs), Ln(lobar CMBs), EPVSs in basal ganglia, EPVSs in centrum semiovale, CSS, and CAA, with each downstream Alzheimer disease plasma biomarker, including p-tau217, GFAP, and NfL, after controlling for age, sex, body mass index status, and *APOE* genotype.

^b^ The presence of CAA was defined as having magnetic resonance imaging features suggestive of probable CAA based on the Boston criteria, version 2.0.

**Figure 2.**
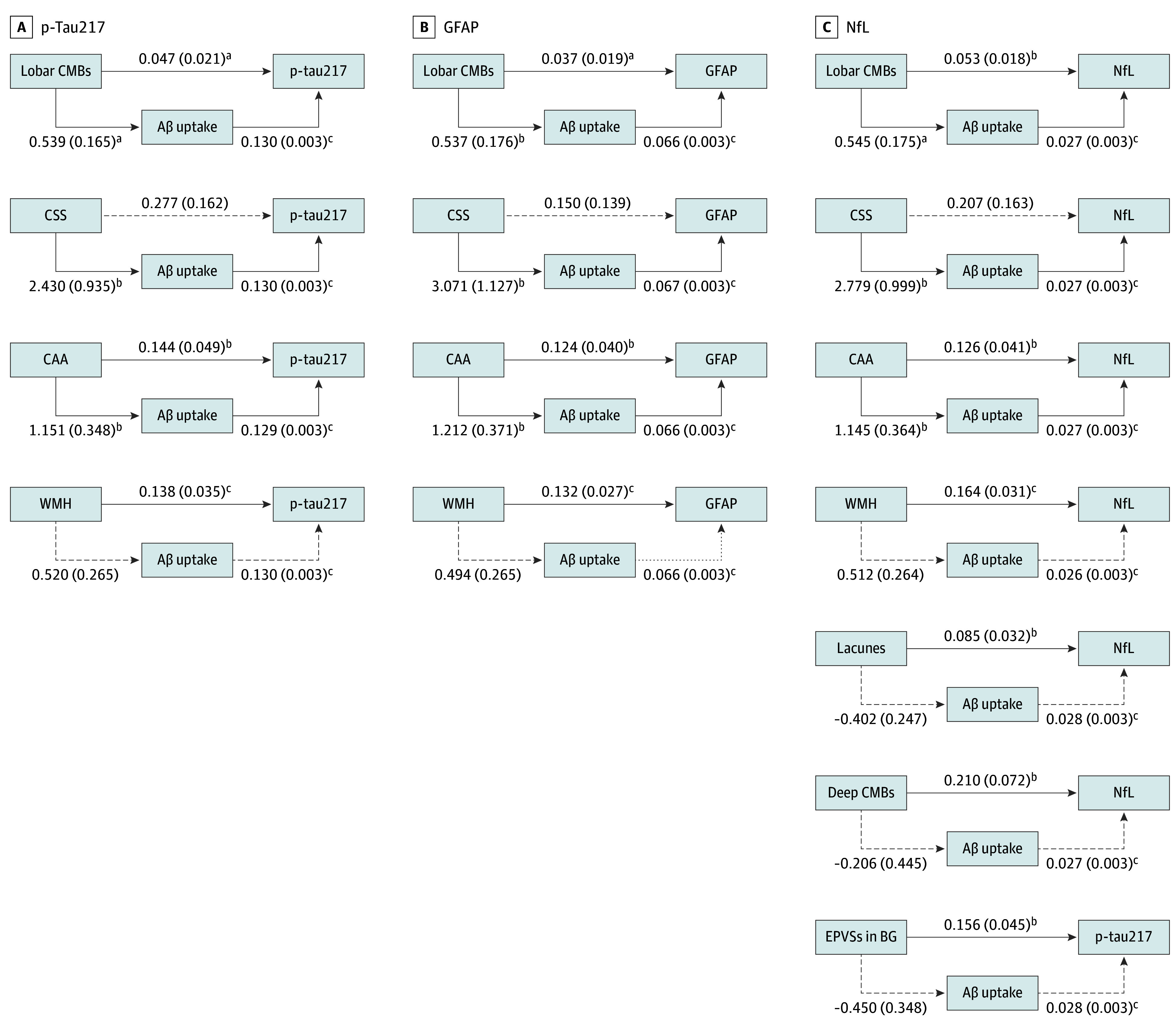
Mediation Analyses Among Cerebral Amyloid Angiopathy (CAA) and Vascular Imaging Markers, Amyloid-β (Aβ) Uptake on Positron Emission Tomography, and Alzheimer Disease Plasma Biomarkers Solid and dashed lines indicate statistically significant and nonsignificant associations, respectively. The β-value (SE) for each association is shown on the corresponding line. Mediation analyses were controlled for age, sex, body mass index status, and *APOE* genotype. The presence of CAA was defined as having magnetic resonance imaging features suggestive of probable CAA based on the Boston criteria, version 2.0. BG indicates basal ganglia; CMB, cerebral microbleed; CSS, cortical superficial siderosis; EPVS, enlarged perivascular space; GFAP, glial fibrillary acidic protein; Ln, natural logarithm; NfL, neurofilament light chain; p-tau217, phosphorylated tau-217; WMH, white matter hyperintensity. ^a^*P* < .05. ^b^*P* < .01. ^c^*P* < .001.

Figure 2 and eTable 2 in Supplement 1 present the associations among CAA and vascular imaging markers, Aβ uptake on PET, and downstream AD plasma biomarkers. The number of lobar CMBs and the presence of CAA were associated with p-tau217, GFAP, and NfL, both with and without the mediation of Aβ uptake (indirect effect: lobar CMBs–p-tau217, 59.8% [β = 0.07 (95% CI, 0.03-0.11)]; lobar CMBs-GFAP, 49.3% [β = 0.04 (95% CI, 0.01-0.06)]; lobar CMBs-NfL, 20.9% [β = 0.01 (95% CI, 0.01-0.03)]; CAA–p-tau217, 50.9% [β = 0.15 (95% CI, 0.06-0.24)]; CAA-GFAP, 39.2% [β = 0.08 (95% CI, 0.03-0.13)]; CAA-NfL, 19.2% [β = 0.03 (95% CI, 0.01-0.05)]). Aβ uptake on PET fully mediated the associations between CSS and plasma biomarkers p-tau217, GFAP, and NfL.

### Associations Among CAA Imaging Makers, Downstream AD Plasma Biomarkers, and Cognitive Changes

**Figure 3.**
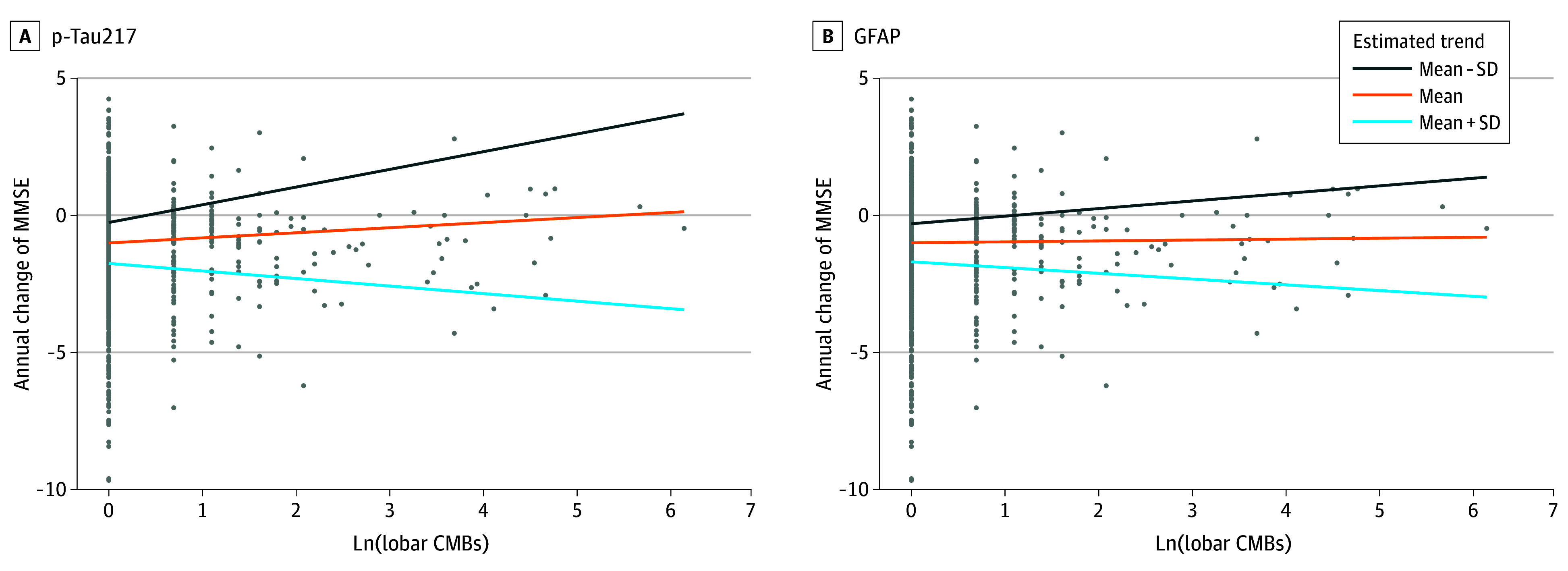
Interaction of Each Alzheimer Disease Plasma Biomarker and Lobar Cerebral Microbleed (CMB) Counts With Annual Mini-Mental State Examination (MMSE) Changes GFAP indicates glial fibrillary acidic protein; Ln, natural logarithm.

A subset of 1381 participants underwent follow-up MMSE assessment. The mean (SD) follow-up period was 4.3 (3.1) years, and the mean (SD) number of MMSE assessments was 3.6 (2.3). There were interactive associations of p-tau217 and lobar CMBs (β = −0.56 [95% CI, −0.79 to −0.34]), and GFAP and lobar CMBs (β = −0.44 [95% CI, −0.70 to −0.17]) with annual MMSE changes. Specifically, high p-tau217 or GFAP levels amplified the association between a higher number of lobar CMBs and greater annual decline in MMSE (Figure 3).

### Subgroup and Sensitivity Analyses

We performed additional subgroup analyses by cognitive stage (no cognitive impairment, mild cognitive impairment, and dementia of the Alzheimer type) with false discovery rate correction to examine whether the associations between CAA and vascular imaging markers and AD plasma biomarkers differed by disease stage. Overall, the direction of the associations observed in the entire cohort remained largely consistent in each subgroup, although some results lost statistical significance in more advanced stages (eFigure 2 in Supplement 1).

We also conducted sensitivity analyses by adding major vascular risk factors (eg, hypertension, diabetes) to our initial covariate. While we observed minor fluctuations, the overall results remained largely consistent (eFigure 3 in Supplement 1).

## Discussion

In this cohort study, we systematically investigated the association of CAA imaging markers with downstream AD plasma biomarkers (p-tau217, GFAP, and NfL) in relation to Aβ uptake on PET in a large Asian cohort. We found that CAA imaging markers, including lobar CMBs and CSS, were associated with p-tau217, GFAP, and NfL levels. Furthermore, Aβ uptake on PET completely or partially mediated the associations between CAA imaging markers and these plasma biomarkers. In contrast, hypertensive arteriosclerotic vascular imaging markers were associated only with NfL levels, regardless of the extent of Aβ uptake on PET. Finally, lobar CMBs in conjunction with p-tau217 and GFAP were synergistically associated with cognitive changes. Taken together, our findings suggest a novel interaction among CAA imaging markers, downstream AD plasma biomarkers, and cognitive decline, emphasizing the importance of understanding the clinical effects of ARIA-like CAA imaging markers in light of upcoming amyloid-targeted therapies.

Our first major finding was that CAA imaging markers are associated with increased downstream AD plasma biomarkers. Additionally, Aβ uptake on PET partially or completely mediated these associations. The completely mediated association may be explained by several factors. Previous studies have reported an association between CAA and Aβ uptake on PET.^44,45^ In addition, p-tau217, GFAP, and NfL levels have been strongly correlated with Aβ uptake.^46,47,48^ A notable finding in our study was that lobar CMBs or the presence of CAA may influence amyloid-independent hyperphosphorylated tau, neuroinflammation, and neurodegeneration. Indeed, previous studies have shown that lobar CMBs are associated with tau pathology independent of amyloid pathology.^49^ The presence of CAA has also been associated with regional production of paired helical filament tau.^50^ Future research that explores the effect of CAA on these downstream AD plasma biomarkers independently of Aβ may provide insight into the mechanisms by which CAA-like ARIA-hemorrhage contributes to the prognosis of patients receiving amyloid-targeted therapy.

The exact reasons for CSS being fully mediated by Aβ uptake, unlike other CAA imaging markers, remain unclear. However, it may be explained by their respective correlations with Aβ uptake on PET. Our study used an Aβ PET that, while related to CAA, primarily reflects neuritic plaques in the brain parenchyma.^51^ Furthermore, CAA may be associated with neuritic plaques, but it may also occur pathologically without evident neuritic plaques.^52,53^ In fact, in our group’s previous research among 65 patients with CAA,^29^ only 43 (66.2%) had Aβ PET–positive results. In contrast, in the current study, 24 of the 25 participants (96.0%) with CSS had an amyloid Klunk CL of 20 or higher, which is markedly higher than the rate observed for other CAA imaging markers. We believe that these differences underscore the distinct pathophysiologic implications of various CAA imaging markers and highlight the importance of considering these nuances in future studies.

Interestingly, the presence of WMHs was associated with p-tau217, GFAP, and NfL levels regardless of the extent of Aβ uptake on PET. Our findings were consistent with previous studies that showed that higher GFAP or NfL levels were associated with greater WMH.^22,23^ While WMHs are commonly associated with cerebral ischemia or reduced blood flow to the brain, they might also result from other processes such as neuroinflammation or neurodegeneration. In neurodegenerative diseases, including AD, the brain’s immune cells may become activated, leading to the release of inflammatory molecules that might contribute to the development of WMH. Furthermore, as brain cells degenerate, the white matter tracts that connect different regions of the brain may become affected, leading to the formation of WMHs.

We also found that hypertensive arteriosclerotic vascular imaging markers, including lacunes, deep CMBs, and EPVSs in the basal ganglia, were associated only with NfL levels independently of brain Aβ burden. Our findings may be explained by several mechanisms, such as degenerative changes in the subcortical regions, including white matter and deep gray matter; secondary neuronal cell body damage and atrophy in the cortex; and concomitant ischemic and degenerative changes in both the cortical and subcortical regions. Results from our study did not distinguish among these 3 different mechanisms.

Subgroup analyses indicated that the overall direction of associations between CAA imaging markers and downstream AD plasma biomarkers was largely consistent across cognitive stages, although some results lost statistical significance in more advanced stages. For instance, the correlation between CAA imaging markers (eg, lobar CMBs and CSS) and p-tau217 remained significant in participants without cognitive impairment and with mild cognitive impairment but not in those with dementia of the Alzheimer type. This correlation may be explained by the overriding influence of robust AD pathologies in dementia of the Alzheimer type, as well as that our plasma biomarkers largely reflect early, soluble AD pathologies. Additionally, dividing the cohort into subgroups reduced the sample size and, hence, the statistical power for each group. We were also unable to perform mediation analyses by cognitive stage due to the small number of participants with CAA imaging markers in each subgroup. Future studies with larger samples or alternative designs will be essential to clarify potential stage-dependent mediation effects.

Another notable finding was that lobar CMBs in conjunction with p-tau217 and GFAP were synergistically associated with cognitive changes. In our group’s previous studies, lobar CMBs were identified as associated with cognitive changes.^4^ Additionally, p-tau217 and GFAP levels have been associated with cognitive changes.^20^ Furthermore, patients with both Aβ-positive uptake on PET and lobar CMBs have shown faster cognitive deterioration than those with lobar CMBs alone.^29^ In our study, we suggest that lobar CMBs may be synergistically associated with cognitive changes along with downstream AD plasma biomarkers indicative of hyperphosphorylated tau and neuroinflammation, in addition to Aβ. Understanding how these factors interact might provide insight into the complex association among CAA-like ARIA-hemorrhage, downstream AD plasma biomarkers, and cognitive impairment in patients receiving amyloid-targeted therapy.

### Limitations

Although our study involved a large Asian population, it had some limitations. First, we did not perform pathologic validation in our participants. Therefore, other vascular pathologies and copathologies, such as frontotemporal lobar degeneration with tau pathology, transactive response DNA-binding protein, argyrophilic grain disease, and hippocampal sclerosis, were not considered. Second, although our plasma biomarkers were developed to reflect downstream AD processes, including hyperphosphorylation of tau, neuroinflammation, and neurodegeneration, they are closely related to Aβ on PET. However, these arguments might be mitigated by CAA imaging markers not being associated with these downstream plasma biomarkers regardless of Aβ uptake on PET. Future studies incorporating plasma biomarkers reflective of more specific downstream markers are needed. Third, since our study was conducted exclusively in a Korean cohort, caution is warranted when extrapolating these results to other racial and ethnic groups. In particular, although some reports have indicated that ARIA frequencies in Asian patients are lower compared with non-Hispanic White patients,^25^ the underlying reasons for this difference remain unclear. Moreover, Asian people tend to have a higher prevalence of small vessel disease, which may further influence the observed associations. Future studies involving diverse racial and ethnic groups are essential to further validate and extend the external applicability of our findings.

## Conclusions

In this cohort study of participants with no cognitive impairment, mild cognitive impairment, or dementia of the Alzheimer type, CAA imaging markers were associated with downstream AD plasma biomarkers in relation to Aβ uptake on PET. Furthermore, CAA imaging markers and these plasma biomarkers were synergistically associated with cognitive decline. Our findings suggest a complex interaction among CAA-like ARIA-hemorrhage, downstream AD plasma biomarkers, and cognitive impairment in patients receiving amyloid-targeted therapy.

## Notes

Supplement 1.
eMethods.

eReferences.
**eTable 1.** Demographic Characteristics of Participants With Missing Data **eTable 2.** Association Among CAA and Vascular Imaging Markers, Aβ Uptake on PET, and Plasma Biomarkers **eFigure 1. **Flow of Participant Selection **eFigure 2.** Forest Plots for Associations of CAA and Vascular Imaging Markers With Plasma Biomarkers Across Cognitive Stages **eFigure 3.** Forest Plots for Associations of CAA and Vascular Imaging Markers With Plasma Biomarkers, Including Additional Analyses With Major Vascular Risk Factors

Supplement 2.
Data Sharing Statement

## References

[1] Sperling RA, Jack CR Jr, Black SE, . Amyloid-related imaging abnormalities in amyloid-modifying therapeutic trials: recommendations from the Alzheimer’s Association Research Roundtable Workgroup. Alzheimers Dement. 2011;7(4):367-385. doi:10.1016/j.jalz.2011.05.2351 21784348 PMC3693547

[2] Hampel H, Elhage A, Cho M, Apostolova LG, Nicoll JAR, Atri A. Amyloid-related imaging abnormalities (ARIA): radiological, biological and clinical characteristics. Brain. 2023;146(11):4414-4424. doi:10.1093/brain/awad188 37280110 PMC10629981

[3] Cenina AR, De Leon J, Tay KY, Wong CF, Kandiah N. Cerebral amyloid angiopathy-related inflammation presenting with rapidly progressive dementia, responsive to IVIg. Alzheimer Dis Assoc Disord. 2015;29(4):347-349. doi:10.1097/WAD.0000000000000084 25710131

[4] Jang YK, Kim HJ, Lee JS, . Distinctive clinical effects of haemorrhagic markers in cerebral amyloid angiopathy. Sci Rep. 2017;7(1):15984. doi:10.1038/s41598-017-16298-1 29167486 PMC5700189

[5] Rissin DM, Kan CW, Campbell TG, . Single-molecule enzyme-linked immunosorbent assay detects serum proteins at subfemtomolar concentrations. Nat Biotechnol. 2010;28(6):595-599. doi:10.1038/nbt.1641 20495550 PMC2919230

[6] Teunissen CE, Verberk IMW, Thijssen EH, . Blood-based biomarkers for Alzheimer’s disease: towards clinical implementation. Lancet Neurol. 2022;21(1):66-77. doi:10.1016/S1474-4422(21)00361-6 34838239

[7] Blennow K, Galasko D, Perneczky R, . The potential clinical value of plasma biomarkers in Alzheimer’s disease. Alzheimers Dement. 2023;19(12):5805-5816. doi:10.1002/alz.13455 37694991

[8] Hansson O, Blennow K, Zetterberg H, Dage J. Blood biomarkers for Alzheimer’s disease in clinical practice and trials. Nat Aging. 2023;3(5):506-519. doi:10.1038/s43587-023-00403-3 37202517 PMC10979350

[9] Leuzy A, Mattsson-Carlgren N, Palmqvist S, Janelidze S, Dage JL, Hansson O. Blood-based biomarkers for Alzheimer’s disease. EMBO Mol Med. 2022;14(1):e14408. doi:10.15252/emmm.202114408 34859598 PMC8749476

[10] Nakamura A, Kaneko N, Villemagne VL, . High performance plasma amyloid-β biomarkers for Alzheimer’s disease. Nature. 2018;554(7691):249-254. doi:10.1038/nature25456 29420472

[11] Karikari TK, Pascoal TA, Ashton NJ, . Blood phosphorylated tau 181 as a biomarker for Alzheimer’s disease: a diagnostic performance and prediction modelling study using data from four prospective cohorts. Lancet Neurol. 2020;19(5):422-433. doi:10.1016/S1474-4422(20)30071-5 32333900

[12] Ashton NJ, Pascoal TA, Karikari TK, . Plasma p-tau231: a new biomarker for incipient Alzheimer’s disease pathology. Acta Neuropathol. 2021;141(5):709-724. doi:10.1007/s00401-021-02275-6 33585983 PMC8043944

[13] Palmqvist S, Janelidze S, Quiroz YT, . Discriminative accuracy of plasma phospho-tau217 for Alzheimer disease vs other neurodegenerative disorders. JAMA. 2020;324(8):772-781. doi:10.1001/jama.2020.12134 32722745 PMC7388060

[14] Mielke MM, Frank RD, Dage JL, . Comparison of plasma phosphorylated tau species with amyloid and tau positron emission tomography, neurodegeneration, vascular pathology, and cognitive outcomes. JAMA Neurol. 2021;78(9):1108-1117. doi:10.1001/jamaneurol.2021.2293 34309632 PMC8314178

[15] Benedet AL, Milà-Alomà M, Vrillon A, ; Translational Biomarkers in Aging and Dementia (TRIAD) Study, Alzheimer’s and Families (ALFA) Study, and BioCogBank Paris Lariboisière Cohort. Differences between plasma and cerebrospinal fluid glial fibrillary acidic protein levels across the Alzheimer disease continuum. JAMA Neurol. 2021;78(12):1471-1483. doi:10.1001/jamaneurol.2021.3671 34661615 PMC8524356

[16] Chatterjee P, Pedrini S, Stoops E, . Plasma glial fibrillary acidic protein is elevated in cognitively normal older adults at risk of Alzheimer’s disease. Transl Psychiatry. 2021;11(1):27. doi:10.1038/s41398-020-01137-1 33431793 PMC7801513

[17] Cicognola C, Janelidze S, Hertze J, . Plasma glial fibrillary acidic protein detects Alzheimer pathology and predicts future conversion to Alzheimer dementia in patients with mild cognitive impairment. Alzheimers Res Ther. 2021;13(1):68. doi:10.1186/s13195-021-00804-9 33773595 PMC8005231

[18] Mattsson N, Andreasson U, Zetterberg H, Blennow K; Alzheimer’s Disease Neuroimaging Initiative. Association of plasma neurofilament light with neurodegeneration in patients with Alzheimer disease. JAMA Neurol. 2017;74(5):557-566. doi:10.1001/jamaneurol.2016.6117 28346578 PMC5822204

[19] Mielke MM, Syrjanen JA, Blennow K, . Plasma and CSF neurofilament light: relation to longitudinal neuroimaging and cognitive measures. Neurology. 2019;93(3):e252-e260. doi:10.1212/WNL.0000000000007767 31182505 PMC6656645

[20] Jang H, Shin D, Yoo H, . Differential roles of Alzheimer’s disease plasma biomarkers in stepwise biomarker-guided diagnostics. Alzheimers Dement. 2025;21(2):e14526. doi:10.1002/alz.14526 39907189 PMC11848384

[21] Chong JR, Chai YL, Yam ATY, . Association of plasma GFAP with elevated brain amyloid is dependent on severity of white matter lesions in an Asian cognitively impaired cohort. Alzheimers Dement (Amst). 2024;16(2):e12576. doi:10.1002/dad2.12576 38605996 PMC11007806

[22] Chong JR, Hilal S, Ashton NJ, . Brain atrophy and white matter hyperintensities are independently associated with plasma neurofilament light chain in an Asian cohort of cognitively impaired patients with concomitant cerebral small vessel disease. Alzheimers Dement (Amst). 2023;15(1):e12396. doi:10.1002/dad2.12396 36994314 PMC10040495

[23] Shir D, Graff-Radford J, Hofrenning EI, . Association of plasma glial fibrillary acidic protein (GFAP) with neuroimaging of Alzheimer’s disease and vascular pathology. Alzheimers Dement (Amst). 2022;14(1):e12291. doi:10.1002/dad2.12291 35252538 PMC8883441

[24] McCarter SJ, Lesnick TG, Lowe VJ, . Association between plasma biomarkers of amyloid, tau, and neurodegeneration with cerebral microbleeds. J Alzheimers Dis. 2022;87(4):1537-1547. doi:10.3233/JAD-220158 35527558 PMC9472282

[25] Chen C, Katayama S, Lee J-H, . Clarity AD: Asian regional analysis of a phase III trial of lecanemab in early Alzheimer’s disease. J Prev Alzheimer Dis. Published online April 5, 2025. doi:10.1016/j.tjpad.2025.10016040189473

[26] Jang H, Shin D, Kim Y, . Korea-Registries to Overcome and Accelerate Dementia Research (K-ROAD): a cohort for dementia research and ethnic-specific insights. Dement Neurocogn Disord. 2024;23(4):212-223. doi:10.12779/dnd.2024.23.4.212 39512701 PMC11538854

[27] World Medical Association. World Medical Association Declaration of Helsinki: ethical principles for medical research involving human subjects. JAMA. 2013;310(20):2191-2194. doi:10.1001/jama.2013.281053 24141714

[28] Kang SH, Kim ME, Jang H, . Amyloid positivity in the Alzheimer/subcortical-vascular spectrum. Neurology. 2021;96(17):e2201-e2211. doi:10.1212/WNL.0000000000011833 33722997

[29] Jang H, Jang YK, Kim HJ, . Clinical significance of amyloid β positivity in patients with probable cerebral amyloid angiopathy markers. Eur J Nucl Med Mol Imaging. 2019;46(6):1287-1298. doi:10.1007/s00259-019-04314-7 30937462

[30] Greenberg SM, Vernooij MW, Cordonnier C, ; Microbleed Study Group. Cerebral microbleeds: a guide to detection and interpretation. Lancet Neurol. 2009;8(2):165-174. doi:10.1016/S1474-4422(09)70013-4 19161908 PMC3414436

[31] Gregoire SM, Chaudhary UJ, Brown MM, . The Microbleed Anatomical Rating Scale (MARS): reliability of a tool to map brain microbleeds. Neurology. 2009;73(21):1759-1766. doi:10.1212/WNL.0b013e3181c34a7d 19933977

[32] Charidimou A, Jäger RH, Fox Z, . Prevalence and mechanisms of cortical superficial siderosis in cerebral amyloid angiopathy. Neurology. 2013;81(7):626-632. doi:10.1212/WNL.0b013e3182a08f2c 23864315

[33] Charidimou A, Boulouis G, Frosch MP, . The Boston criteria version 2.0 for cerebral amyloid angiopathy: a multicentre, retrospective, MRI-neuropathology diagnostic accuracy study. Lancet Neurol. 2022;21(8):714-725. doi:10.1016/S1474-4422(22)00208-3 35841910 PMC9389452

[34] Noh Y, Lee Y, Seo SW, . A new classification system for ischemia using a combination of deep and periventricular white matter hyperintensities. J Stroke Cerebrovasc Dis. 2014;23(4):636-642. doi:10.1016/j.jstrokecerebrovasdis.2013.06.002 23867045

[35] Duering M, Biessels GJ, Brodtmann A, . Neuroimaging standards for research into small vessel disease-advances since 2013. Lancet Neurol. 2023;22(7):602-618. doi:10.1016/S1474-4422(23)00131-X 37236211

[36] Doubal FN, MacLullich AM, Ferguson KJ, Dennis MS, Wardlaw JM. Enlarged perivascular spaces on MRI are a feature of cerebral small vessel disease. Stroke. 2010;41(3):450-454. doi:10.1161/STROKEAHA.109.564914 20056930

[37] Wardlaw JM, Smith EE, Biessels GJ, ; Standards for Reporting Vascular Changes on Neuroimaging (STRIVE v1). Neuroimaging standards for research into small vessel disease and its contribution to ageing and neurodegeneration. Lancet Neurol. 2013;12(8):822-838. doi:10.1016/S1474-4422(13)70124-8 23867200 PMC3714437

[38] Klunk WE, Koeppe RA, Price JC, . The Centiloid Project: standardizing quantitative amyloid plaque estimation by PET. Alzheimers Dement. 2015;11(1):1-15.e1-4. doi:10.1016/j.jalz.2014.07.003 25443857 PMC4300247

[39] Rowe CC, Doré V, Jones G, . ^18^F-florbetaben PET beta-amyloid binding expressed in centiloids. Eur J Nucl Med Mol Imaging. 2017;44(12):2053-2059. doi:10.1007/s00259-017-3749-6 28643043 PMC5656696

[40] Battle MR, Pillay LC, Lowe VJ, . Centiloid scaling for quantification of brain amyloid with [^18^F]flutemetamol using multiple processing methods. EJNMMI Res. 2018;8(1):107. doi:10.1186/s13550-018-0456-7 30519791 PMC6281542

[41] Lee EH, Kang SH, Shin D, . Plasma Alzheimer’s disease biomarker variability: amyloid-independent and amyloid-dependent factors. Alzheimers Dement. 2025;21(1):e14368. doi:10.1002/alz.14368 39535473 PMC11782842

[42] Cho SH, Jang M, Ju H, Kang MJ, Yun JM, Yun JW. Association of late-life body mass index with the risk of Alzheimer disease: a 10-year nationwide population-based cohort study. Sci Rep. 2022;12(1):15298. doi:10.1038/s41598-022-19696-2 36097042 PMC9468036

[43] Kim H, Kim C, Seo SW, . Association between body mass index and cortical thickness: among elderly cognitively normal men and women. Int Psychogeriatr. 2015;27(1):121-130. doi:10.1017/S1041610214001744 25263181

[44] Farid K, Charidimou A, Baron JC. Amyloid positron emission tomography in sporadic cerebral amyloid angiopathy: a systematic critical update. Neuroimage Clin. 2017;15:247-263. doi:10.1016/j.nicl.2017.05.002 28560150 PMC5435601

[45] Charidimou A, Farid K, Baron JC. Amyloid-PET in sporadic cerebral amyloid angiopathy: a diagnostic accuracy meta-analysis. Neurology. 2017;89(14):1490-1498. doi:10.1212/WNL.0000000000004539 28855406

[46] Therriault J, Vermeiren M, Servaes S, . Association of phosphorylated tau biomarkers with amyloid positron emission tomography vs tau positron emission tomography. JAMA Neurol. 2023;80(2):188-199. doi:10.1001/jamaneurol.2022.4485 36508198 PMC9856704

[47] Rauchmann BS, Schneider-Axmann T, Perneczky R. Associations of longitudinal plasma p-tau181 and NfL with tau-PET, Aβ-PET and cognition. J Neurol Neurosurg Psychiatry. 2021;92(12):1289-1295. doi:10.1136/jnnp-2020-325537 34187867 PMC8606440

[48] Pereira JB, Janelidze S, Smith R, . Plasma GFAP is an early marker of amyloid-β but not tau pathology in Alzheimer’s disease. Brain. 2021;144(11):3505-3516. doi:10.1093/brain/awab223 34259835 PMC8677538

[49] Chiang GC, Cruz Hernandez JC, Kantarci K, Jack CR Jr, Weiner MW; Alzheimer’s Disease Neuroimaging Initiative. Cerebral microbleeds, CSF p-tau, and cognitive decline: significance of anatomic distribution. AJNR Am J Neuroradiol. 2015;36(9):1635-1641. doi:10.3174/ajnr.A4351 26228889 PMC4729288

[50] Kim HJ, Cho H, Werring DJ, . 18F-AV-1451 PET imaging in three patients with probable cerebral amyloid angiopathy. J Alzheimers Dis. 2017;57(3):711-716. doi:10.3233/JAD-161139 28282808

[51] Seo SW, Ayakta N, Grinberg LT, . Regional correlations between [^11^C]PIB PET and post-mortem burden of amyloid-beta pathology in a diverse neuropathological cohort. Neuroimage Clin. 2016;13:130-137. doi:10.1016/j.nicl.2016.11.008 27981028 PMC5144753

[52] Attems J. Sporadic cerebral amyloid angiopathy: pathology, clinical implications, and possible pathomechanisms. Acta Neuropathol. 2005;110(4):345-359. doi:10.1007/s00401-005-1074-9 16170565

[53] Ellis RJ, Olichney JM, Thal LJ, . Cerebral amyloid angiopathy in the brains of patients with Alzheimer’s disease: the CERAD experience, part XV. Neurology. 1996;46(6):1592-1596. doi:10.1212/WNL.46.6.1592 8649554

