# Supplementary material for: Cerebral Amyloid Angiopathy and Downstream Alzheimer Disease Plasma Biomarkers
Source: JAMA Netw Open. 2025 May 9;8(5):e258842. doi: 10.1001/jamanetworkopen.2025.8842 (PMC12065043; doi:10.1001/jamanetworkopen.2025.8842)
Supplement: Supplement 1. — eMethods. eReferences. eTable 1. Demographic Characteristics of Participants With Missing Data eTable 2. Association Among CAA and Vascular Imaging Markers, Aβ Uptake on PET, and Plasma Biomarkers eFigure 1. Flow of Participant Selection eFigure 2. Forest Plots for Associations of CAA and Vascular Imaging Markers With Plasma Biomarkers Across Cognitive Stages eFigure 3. Forest Plots for Associations of CAA and Vascular Imaging Markers With Plasma Biomarkers, Including Additional Analyses With Major Vascular Risk Factors [file jamanetwopen-e258842-s001.pdf]

Supplemental Online Content

Kang SH, Lee EH, Kim YJ, et al. Cerebral amyloid angiopathy and downstream Alzheimer disease plasma biomarkers. *JAMA Netw Open*. 2025;8(5):e258842. doi:10.1001/jamanetworkopen.2025.8842

eMethods

eReferences

eTable 1. Demographic Characteristics of Participants With Missing Data

eTable 2. Association Among CAA and Vascular Imaging Markers, Aβ Uptake on PET, and Plasma Biomarkers

eFigure 1. Flow of Participant Selection

eFigure 2. Forest Plots for Associations of CAA and Vascular Imaging Markers With Plasma Biomarkers Across Cognitive Stages

eFigure 3. Forest Plots for Associations of CAA and Vascular Imaging Markers With Plasma Biomarkers, Including Additional Analyses With Major Vascular Risk Factors

This supplemental material has been provided by the authors to give readers additional information about their work.

© 2025 Kang SH et al. *JAMA Network Open*.

eMethods

Diagnostic Criteria

All cognitively unimpaired participants met the following criteria: (1) no objective cognitive impairment in any cognitive domain on a comprehensive neuropsychological test battery (scores above at least 1.0 SD of age-adjusted norms on any cognitive test); and (2) independence in activities of daily living. All participants with mild cognitive impairment met the criteria for MCI with the following modifications[1,2]: (1) subjective cognitive complaints by the participants or caregivers; (2) objective memory impairment below 1.0 SD on the verbal or visual memory tests; (3) no significant impairment in activities of daily living; and (4) a non-demented status. All participants with dementia of the Alzheimer's type met the National Institute on Aging and Alzheimer's Association (NIA-AA) diagnostic criteria.[3]

Brain Magnetic Resonance Imaging Acquisition

All participants underwent brain MRI, including three-dimensional T1 turbo field echo images, fluid-attenuated inversion recovery (FLAIR), and T2-weighted gradient-echo images (T2-GRE) using the same type of 3.0T MRI scanner (Philips 3.0T Achieva; Philips Healthcare, Andover, MA, United States) at Samsung Medical Center. The 3D T1 images were acquired with the following parameters: sagittal slice thickness 1.0 mm, contiguous slices with 50% overlap, repetition time (TR) 9.9 ms, echo time (TE) 4.6 ms, flip angle 8°, and matrix size 240 × 240 pixels, reconstructed to 480 × 480 over a field of view of 240 mm. The FLAIR images were acquired with the following parameters: axial slice thickness 2 mm, no gap, TR 11,000 ms, TE 125 ms, flip angle 90°, and matrix size 512 × 512 pixels.The T2-GRE images were acquired with the following parameters: axial slice thickness 5.0 mm, interslice

© 2025 Kang SH et al. *JAMA Network Open*.

thickness 2 mm, TR 669 ms, TE 16 ms, flip angle 18°, and matrix size 560 × 560 pixels.[4]

White Matter Hyperintensity Visual Rating

The white matter hyperintensity (WMH) visual rating scale proposed by the Clinical Research Center for Dementia of South Korea (CREDOS) was used to investigate WMH in the deep subcortical and periventricular regions in FLAIR images by an experienced neurologist, as reported in the literature.[5] Briefly, deep WMHs were classified as D1 (<10 mm), D2 (10–25 mm), or D3 (≥25 mm) based on the longest diameter of the lesions. Periventricular WMHs were classified as P1 (cap and band <5 mm), P2 (5–10 mm), or P3 (cap or band ≥10 mm) based on the maximum length measured perpendicular (cap) and horizontal (band) to the ventricle. The combination of these D and P ratings yielded 9 cells, and the overall WMH severity (minimal, moderate, and severe) was defined based on the following combination of D and P ratings: minimal (D1P1, D1P2), moderate (D1P3, D2P1, D2P2, D2P3, D3P1, D3P2), and severe (D3P3).

Aβ PET Quantification Using Centiloid Values

We followed the centiloid (CL) process as described by Klunk et al.[6] There are three steps to obtain CL values: (1) pre-processing of PET images, (2) determination of CL global cortical target volume of interest (CTX VOI), and (3) conversion of standardized uptake value ratio (SUVR) to CL values. First, to pre-process the Aβ PET images, PET images were co-registered to each participant's MR image and then normalized to a T1-weighted MNI-152 template using the SPM8 unified segmentation method. We used T1-weighted MR image correction with the N3

© 2025 Kang SH et al. *JAMA Network Open*.

algorithm for intensity nonuniformities, without applying corrections to the PET images for brain atrophy or partial volume effects. Second, we used the standard CL global CTX VOI from the Global Alzheimer's Association Interactive Network (GAAIN) website. Finally, the SUVR values of the global CTX VOI were converted to CL units. To acquire CL units, we first calculated the SUVR standard using the whole cerebellum as a reference, and then calculated the FBB and FMM CL standard values using the CL transformation equation derived from previous studies on FBB (FBB CLstandard = 153.4 × FBB SUVRstandard - 154.9)[7] and FMM (FMM CLstandard =121.42 × FMM SUVRstandard - 121.16).[8]

© 2025 Kang SH et al. *JAMA Network Open*.

eReferences

1. Petersen RC. Clinical practice. Mild cognitive impairment. *N Engl J Med*. 2011;364(23):2227-2234. doi:10.1056/NEJMcp0910237

2. Jeong HJ, Lee H, Lee S-Y, et al. [18F]THK5351 PET Imaging in Patients with Mild Cognitive Impairment. *J Clin Neurol*. 2020;16(2):202-214.

3. McKhann GM, Knopman DS, Chertkow H, et al. The diagnosis of dementia due to Alzheimer's disease: recommendations from the National Institute on Aging-Alzheimer's Association workgroups on diagnostic guidelines for Alzheimer's disease. *Alzheimers Dement*. 2011;7(3):263-269. doi:10.1016/j.jalz.2011.03.005

4. Jang H, Jang YK, Kim HJ, et al. Clinical significance of amyloid beta positivity in patients with probable cerebral amyloid angiopathy markers. *Eur J Nucl Med Mol Imaging*. 2019;46(6):1287-1298. doi:10.1007/s00259-019-04314-7

5. Noh Y, Lee Y, Seo SW, et al. A new classification system for ischemia using a combination of deep and periventricular white matter hyperintensities. *J Stroke Cerebrovasc Dis*. 2014;23(4):636-642. doi:10.1016/j.jstrokecerebrovasdis.2013.06.002

6. Klunk WE, Koeppe RA, Price JC, et al. The Centiloid Project: standardizing quantitative amyloid plaque estimation by PET. *Alzheimers Dement*. 2015;11(1):1-15 e11-14. doi:10.1016/j.jalz.2014.07.003

7. Rowe CC, Doré V, Jones G, et al. 18F-Florbetaben PET beta-amyloid binding expressed in Centiloids. *European Journal of Nuclear Medicine and Molecular Imaging*. 2017;44(12):2053-2059. doi:10.1007/s00259-017-3749-6

8. Battle MR, Pillay LC, Lowe VJ, et al. Centiloid scaling for quantification of brain amyloid with [18F]flutemetamol using multiple processing methods. *EJNMMI Research*. 2018;8(1) doi:10.1186/s13550-018-0456-7

© 2025 Kang SH et al. *JAMA Network Open*.

eTable 1. Demographic Characteristics of Participants With Missing Data

	Klunk centiloid values			Follow-up MMSE scores		
**Study Population (N=1708)**	**Data available (N=1,634)**	**Data not available (N=74)**	***P***	**Data available (N=1,381)**	**Data not available (N=327)**	***P***
Demographics						
Age, years	71.2 (8.4)	72.2 (7.0)	0.31	71.7 (8.2)	69.0 (8.8)	<0.001
Female	1005 (61.5)	39 (52.7)	0.13	838 (60.7)	206 (63.0)	0.44
Male	629 (38.5)	35 (47.3)		543 (39.3)	121 (37.0)	
Education, years	11.4 (4.7)	11.7 (4.0)	0.67	11.4 (4.7)	11.7 (4.9)	0.22
APOE genotype						
ε3ε3	809 (49.6)	40 (54.1)	0.71	668 (48.4)	181 (55.5)	0.01
ε4 carriers	709 (43.4)	30 (40.5)		621 (45.0)	118 (36.2)	
ε2 carriers	114 (7.0)	4 (5.4)		91 (6.6)	27 (8.3)	
BMI status[a]						
Normal weight	1112 (68.1)	37 (50.0)	0.002	927 (67.1)	222 (67.9)	0.44
Obesity	462 (28.3)	35 (47.3)		400 (29.0)	97 (29.7)	
Underweight/	60 (3.7)	2 (2.7)		54 (3.9)	8 (2.4)	
Vascular risk factors						
Hypertension	758 (46.4)	42 (56.8)	0.08	648 (46.9)	152 (46.5)	0.89
Diabetes mellitus	356 (21.8)	14 (18.9)	0.56	300 (21.7)	70 (21.4)	0.90
CAA and vascular imaging markers						
Lobar CMBs, number	1.6 (15.5)	2.5 (12.0)	0.64	1.9 (17.0)	0.6 (3.1)	0.17
Presence of cSS, n (%)	22 (1.3)	3 (4.2)	0.05	23 (1.7)	2 (0.6)	0.15
Presence of CAA[b], n (%)	160 (9.8)	6 (8.3)	0.68	146 (10.6)	20 (6.1)	0.01
Presence of WMH, n (%)	372 (22.8)	19 (25.7)	0.56	320 (23.2)	71 (21.7)	0.57

© 2025 Kang SH et al. *JAMA Network Open*.

Lacunes, number	0.3 (1.0)	0.4 (1.0)	0.58	0.3 (1.1)	0.3 (0.9)	0.94
Deep CMBs, number	0.1 (1.2)	0.1 (0.5)	0.95	0.1 (0.7)	0.2 (2.2)	0.06
Presence of EPVS in BG, n (%)	156 (9.5)	9 (12.2)	0.46	133 (9.6)	32 (9.8)	0.93
Presence of EPVS in CSO, n (%)	422 (25.8)	22 (29.7)	0.45	352 (25.5)	92 (28.1)	0.33

Values are presented as mean (standard deviation) or number (%).

[a] <18.5 kg/m2, underweight; 18.5-24.9 kg/m2, normal weight; and >25 kg/m2, obese.

[b] The presence of CAA was defined as having MRI features suggestive of probable CAA based on Boston criteria version 2.0.

Abbreviations: BMI, body mass index; BG, basal ganglia; CAA, cerebral amyloid angiopathy; CMBs, cerebral microbleeds; cSS, cortical superficial siderosis; CSO, centrum semiovale; CU, cognitively unimpaired; DAT, dementia of the Alzheimer's type; EPVS, enlarged perivascular space; MCI, mild cognitive impairment; MMSE, Mini-Mental Status examination; WMH, white matter hyperintensity.

© 2025 Kang SH et al. *JAMA Network Open*.

eTable 2. Association Among CAA and Vascular Imaging Markers, Aβ Uptake on PET, and Plasma Biomarkers

		p-tau217			GFAP			NFL		
		β (95% CI)	*P*	% indirect[a]	β (95% CI)	*P*	% indirect[a]	β (95% CI)	*P*	% indirect[a]
**Number of lobar MBs**										
Indirect	Lobar CMBs→ Aβ→PBM	0.07 (0.03 – 0.11)[b]	0.001	59.8	0.04 (0.01 – 0.06)[b]	0.003	49.3	0.01 (0.01 – 0.03)[b]	0.003	20.9
Direct	Lobar CMBs →PBM	0.05 (0.01 – 0.09)	0.03		0.04 (0.00 – 0.07)	0.045		0.05 (0.02 – 0.09)	0.004	
Total		0.12 (0.06 – 0.17)	<0.001		0.07 (0.03 – 0.11)	<0.001		0.07 (0.03 – 0.10)	<0.001	
**Presence of cSS**										
Indirect	cSS→Aβ→PBM	0.32 (0.05 – 0.54)[b]	0.009	NA	0.21 (0.07 – 0.37)[b]	0.009	NA	0.08 (0.03 – 0.14)[b]	0.01	NA
Direct	cSS→PBM	0.28 (–0.04 – 0.60)	0.09		0.15 (–0.12 – 0.42)	0.28		0.21 (–0.11 – 0.53)	0.2	
Total		0.59 (0.27 – 0.92)	<0.001		0.35 (0.01 – 0.70)	0.045		0.28 (–0.05 – 0.62)	0.1	
**Presence of CAA**										
Indirect	CAA→Aβ→PBM	0.15 (0.06 – 0.24)[b]	0.001	50.9	0.08 (0.03 – 0.13)[b]	0.002	39.2	0.03 (0.01 – 0.05)[b]	0.003	19.2
Direct	CAA→PBM	0.14 (0.05 – 0.24)	0.003		0.12 (0.05 – 0.20)	0.002		0.13 (0.05 – 0.21)	0.002	
Total		0.29 (0.18 – 0.41)	<0.001		0.20 (0.12 – 0.29)	<0.001		0.16 (0.07 – 0.24)	<0.001	
**Presence of WMH**										
Indirect	WMH→Aβ→PBM	0.07 (–0.00 – 0.13)[b]	0.05	NA	0.03 (0.00 – 0.07)[b]	0.07	NA	0.01 (0.00 – 0.03)[b]	0.06	NA
Direct	WMH→PBM	0.14 (0.07 – 0.21)	<0.001		0.13 (0.08 – 0.19)	<0.001		0.16 (0.10 – 0.23)	<0.001	
Total		0.21 (0.12 – 0.30)	<0.001		0.17 (0.11 – 0.22)	<0.001		0.18 (0.12 – 0.24)	<0.001	

Mediation analyses were performed using each CSVD marker, which affects plasma biomarkers, in the linear regression models as a predictor. Here, Aβ uptake on PET served as a mediator, with plasma biomarkers as an outcome, controlling for age, sex, BMI status, and APOE genotype.

[a]% of indirect effect. These values were not applicable when the relationship was fully mediated by Aβ uptake (100%) or when the indirect effects were insignificant (0%)."

[b]Bootstrap 95% CI

Abbreviations: Aβ, β-amyloid uptake; BG, basal ganglia; CAA, cerebral amyloid angiopathy; CI, confidence interval; CMBs, cerebral microbleeds; CSVD, cerebral small vessel disease; cSS, cortical superficial siderosis; EPVS, enlarged perivascular space; GFAP, glial fibrillary acidic protein; NA, not applicable; NFL, neurofilament light; PBM, plasma biomarker; WMH, white matter hyperintensity.

© 2025 Kang SH et al. *JAMA Network Open*.

Figure 1. Flow of Participant Selection

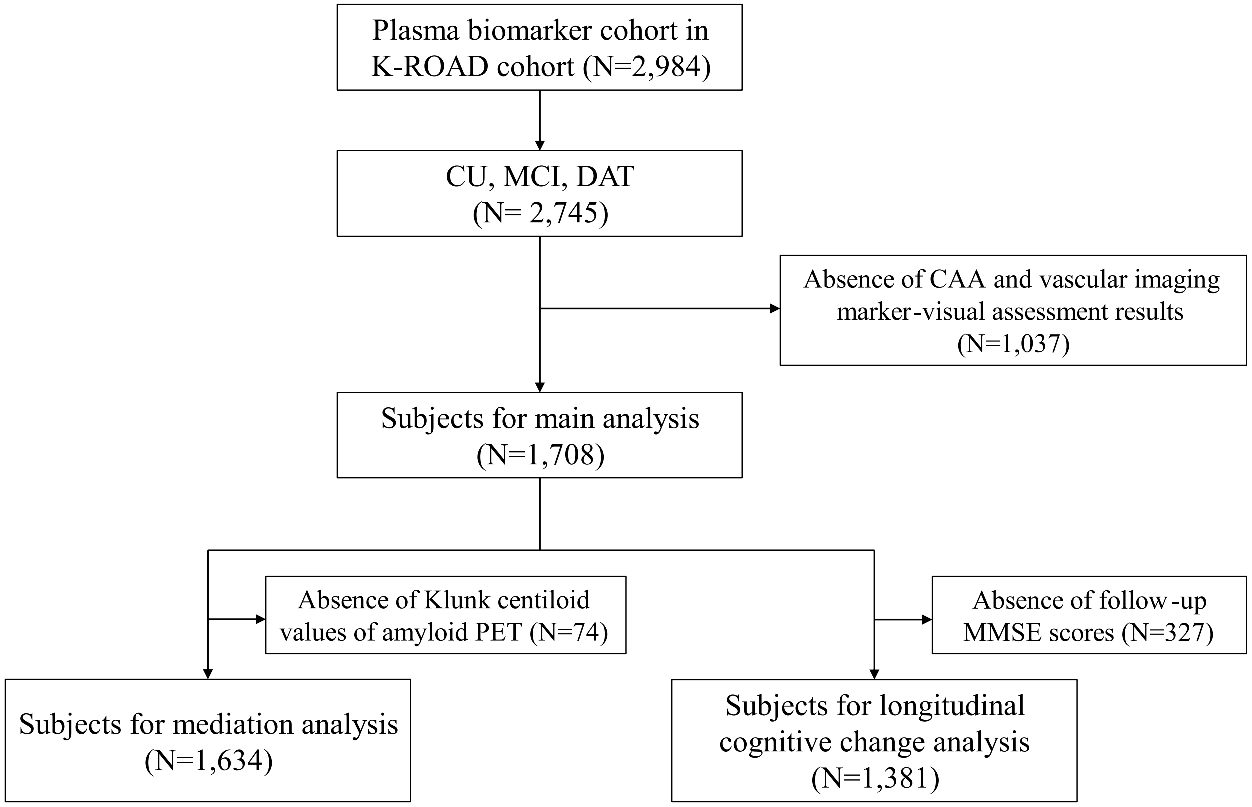

We recruited CU, MCI, and DAT participants from 2,984 individuals in the plasma biomarker cohort of the K-ROAD cohort. After excluding 1,037 individuals without visual assessment results of CAA and vascular imaging markers, the main analysis was conducted on 1,708 individuals. Of these 1,708 individuals, 74 without Klunk centiloid values of amyloid PET were excluded from the mediation analysis, and 166 without follow-up MMSE scores were excluded from the longitudinal cognitive change analysis.
Abbreviations: CAA, cerebral amyloid angiopathy; CU, cognitively unimpaired individuals; DAT, dementia of the Alzheimer's type; K-ROAD, Korea-Registries to Overcome and Accelerate Dementia Research; MCI, mild cognitive impairment; MMSE, Mini-Mental Status examination; PET, positron emission tomography.

© 2025 Kang SH et al. *JAMA Network Open*.

eFigure 2. Forest Plots for Associations of CAA and Vascular Imaging Markers With Plasma Biomarkers Across Cognitive Stages

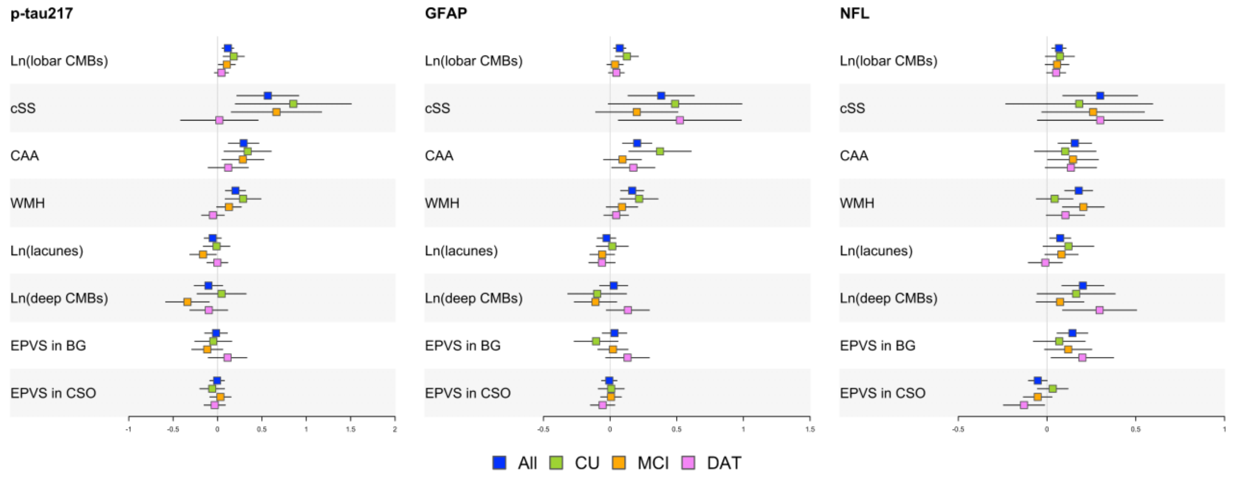

These forest plots display regression coefficients and 95% confidence intervals for the associations between cerebral amyloid angiopathy (CAA)/vascular imaging markers (lobar CMBs, cSS, CAA, WMH, lacunes, deep CMBs, EPVS in the BG, and EPVS in the CSO) and three plasma biomarkers (p-tau217, GFAP, and NFL). The results are presented for all participants (blue) as well as separately for those with cognitively unimpaired (CU, yellow), mild cognitive impairment (MCI, orange), and dementia of the Alzheimer's type (DAT, purple).
Abbreviations: Aβ, amyloid-β; BMI, body mass index; BG, basal ganglia; CAA, cerebral amyloid angiopathy; CMBs, cerebral microbleeds; cSS, cortical superficial siderosis; CSO, centrum semiovale; CU, cognitively unimpaired individuals; DAT, dementia of the Alzheimer's type; EPVS, enlarged perivascular space; GFAP, glial fibrillary acidic protein; MCI, mild cognitive impairment; NFL, neurofilament light; WMH, white matter hyperintensity.

© 2025 Kang SH et al. *JAMA Network Open*.

eFigure 3. Forest Plots for Associations of CAA and Vascular Imaging Markers With Plasma Biomarkers, Including Additional Analyses With Major Vascular Risk Factors

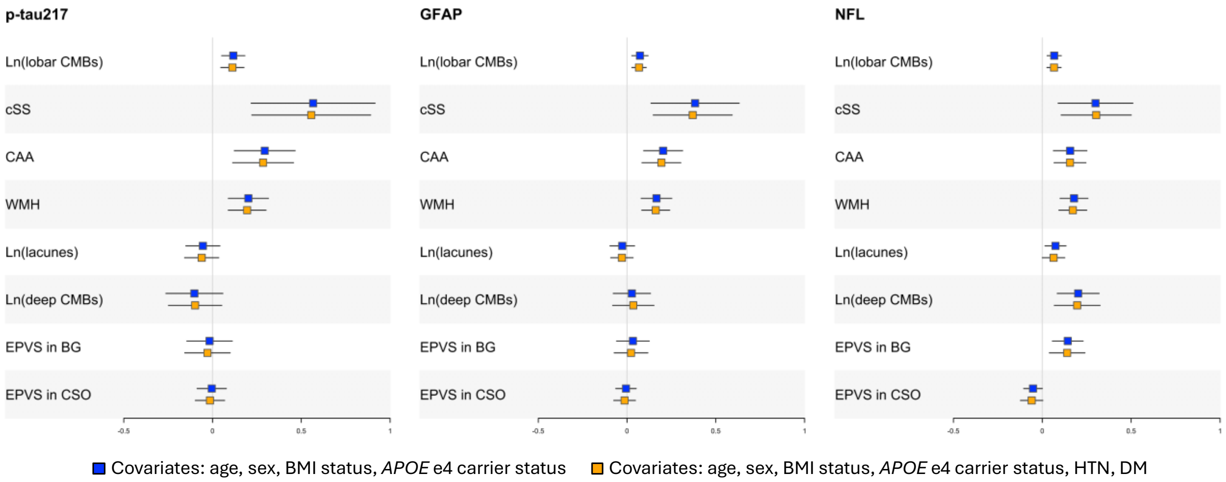

The forest plots show effect sizes (95% CIs) for the associations between CSVD markers (lobar CMBs, cSS, CAA, WMH, lacunes, deep CMBs, and EPVS) and plasma biomarkers (p-tau217, GFAP, NFL). Two models were tested: one adjusted for age, sex, BMI status and APOE ε4 carrier status (blue bars), and another that added major vascular risk factors (hypertension, diabetes) to these initial covariates (orange bars). In this study, BMI status was stratified as follows: <18.5 kg/m2, underweight; 18.5-24.9 kg/m2, normal weight; and >25 kg/m2, obese.

Abbreviations: Aβ, amyloid-β; BMI, body mass index; BG, basal ganglia; CAA, cerebral amyloid angiopathy; CMBs, cerebral microbleeds; cSS, cortical superficial siderosis; CSO, centrum semiovale; DM, diabetes mellitus; EPVS, enlarged perivascular space; GFAP, glial fibrillary acidic protein; HTN, hypertension; NFL, neurofilament light; WMH, white matter hyperintensity.

© 2025 Kang SH et al. *JAMA Network Open*.
