# Supplementary material for: Cerebral Amyloid Angiopathy and Downstream Alzheimer Disease Plasma Biomarkers
Source: JAMA Netw Open. 2025 May 9;8(5):e258842. doi: 10.1001/jamanetworkopen.2025.8842 (PMC12065043; doi:10.1001/jamanetworkopen.2025.8842)
Supplement: Supplement 2. — Data Sharing Statement [file jamanetwopen-e258842-s002.pdf]

Data Sharing Statement

Kang. Cerebral Amyloid Angiopathy and Downstream Alzheimer Disease Plasma Biomarkers. *JAMA Netw Open*. Published May 09, 2025. doi:10.1001/jamanetworkopen.2025.8842

Data
Data available: Yes
Data types: Deidentified participant data
How to access data: Anonymized data for our analyses presented in the present report are available from the corresponding authors upon request.
When available: With publication

Supporting Documents
Document types: None

Additional Information
Who can access the data: researchers whose proposed use of the data has been approved
Types of analyses: for any purpose
Mechanisms of data availability: with investigator support
